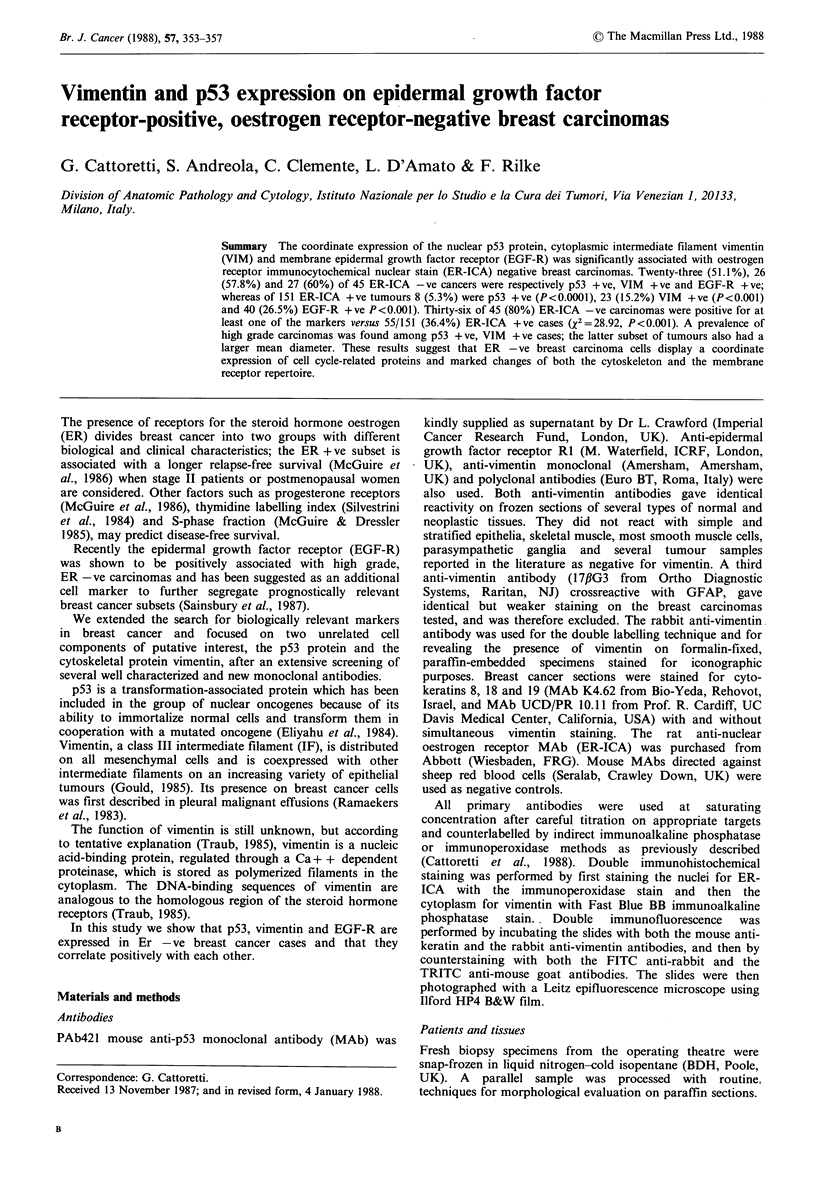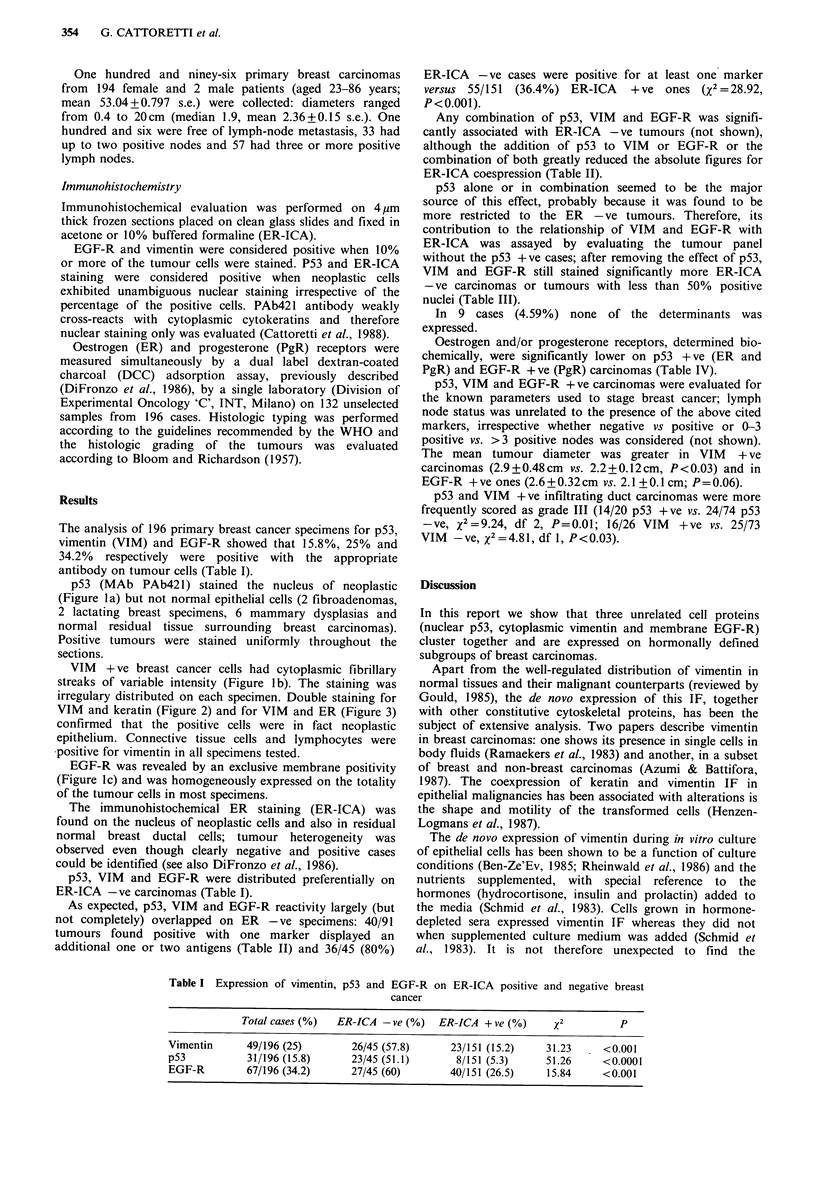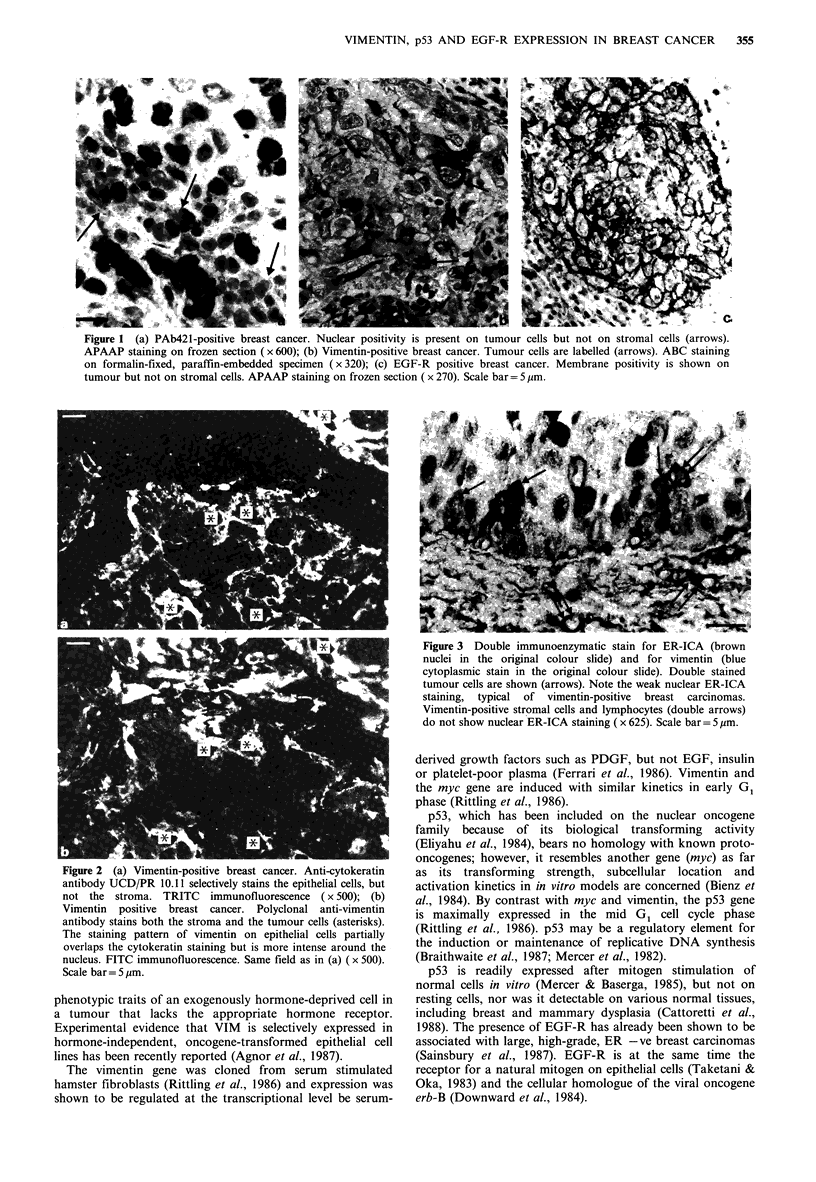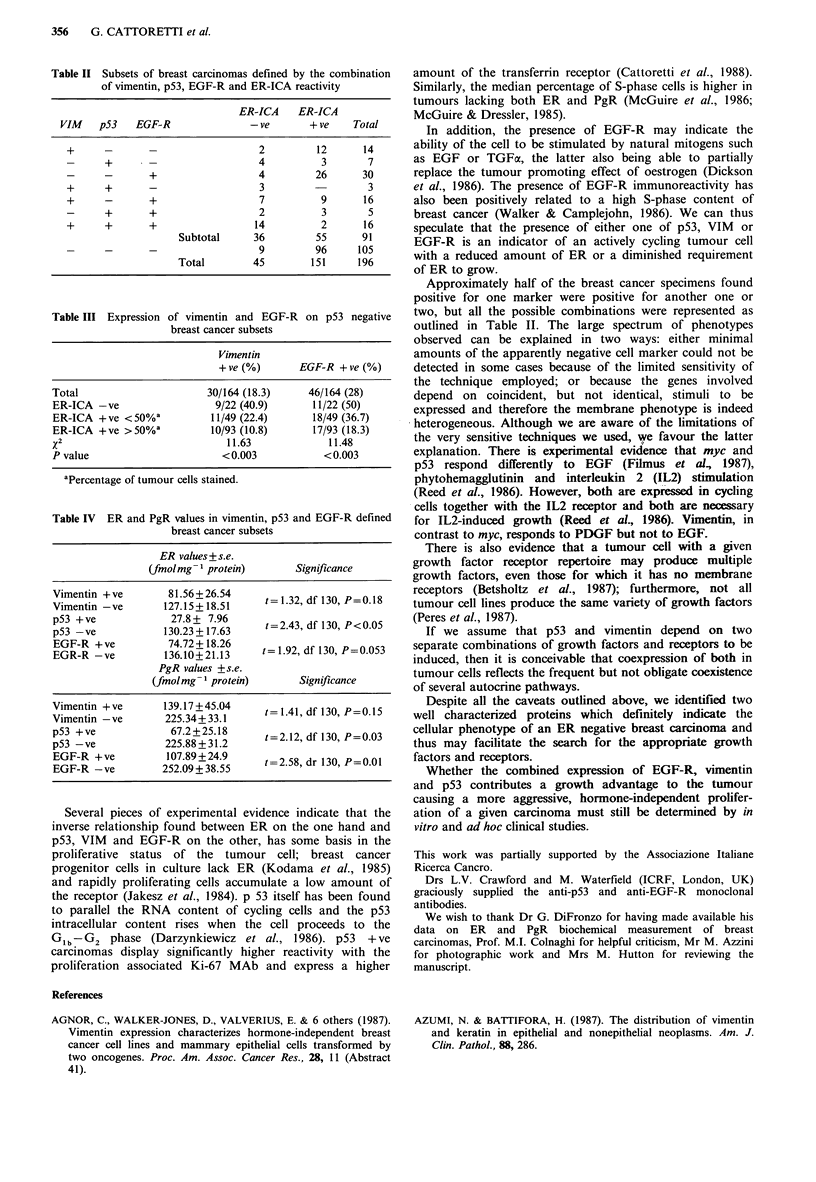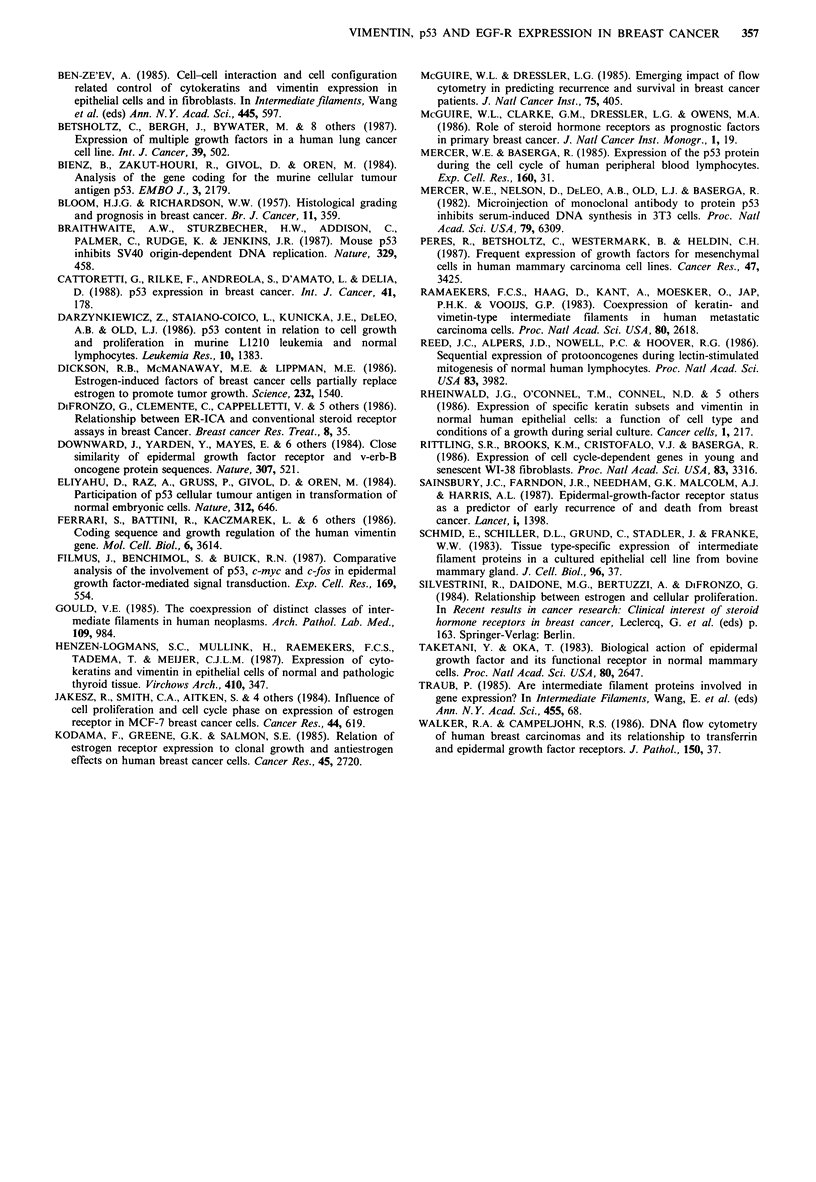# Vimentin and p53 expression on epidermal growth factor receptor-positive, oestrogen receptor-negative breast carcinomas.

**DOI:** 10.1038/bjc.1988.81

**Published:** 1988-04

**Authors:** G. Cattoretti, S. Andreola, C. Clemente, L. D'Amato, F. Rilke

**Affiliations:** Division of Anatomic Pathology and Cytology, Istituto Nazionale per lo Studio e la Cura dei Tumori, Milano, Italy.

## Abstract

**Images:**


*Br. J. Cancer* (1988), **57**, 353–357
© The Macmillan Press Ltd., 1988

### Vimentin and p53 expression on epidermal growth factor receptor-positive, oestrogen receptor-negative breast carcinomas

G. Cattoretti, S. Andreola, C. Clemente, L. D'Amato & F. Rilke

*Division of Anatomic Pathology and Cytology, Istituto Nazionale per lo Studio e la Cura dei Tumori, Via Venezian 1, 20133, Milano, Italy.*

**Summary** The coordinate expression of the nuclear p53 protein, cytoplasmic intermediate filament vimentin (VIM) and membrane epidermal growth factor receptor (EGF-R) was significantly associated with oestrogen receptor immunocytochemical nuclear stain (ER-ICA) negative breast carcinomas. Twenty-three (51.1%), 26 (57.8%) and 27 (60%) of 45 ER-ICA −ve cancers were respectively p53 +ve, VIM +ve and EGF-R +ve; whereas of 151 ER-ICA +ve tumours 8 (5.3%) were p53 +ve ($P<0.0001$), 23 (15.2%) VIM +ve ($P<0.001$) and 40 (26.5%) EGF-R +ve $P<0.001$). Thirty-six of 45 (80%) ER-ICA −ve carcinomas were positive for at least one of the markers *versus* 55/151 (36.4%) ER-ICA +ve cases ($\chi^2=28.92$, $P<0.001$). A prevalence of high grade carcinomas was found among p53 +ve, VIM +ve cases; the latter subset of tumours also had a larger mean diameter. These results suggest that ER −ve breast carcinoma cells display a coordinate expression of cell cycle-related proteins and marked changes of both the cytoskeleton and the membrane receptor repertoire.

The presence of receptors for the steroid hormone oestrogen (ER) divides breast cancer into two groups with different biological and clinical characteristics; the ER +ve subset is associated with a longer relapse-free survival (McGuire *et al.*, 1986) when stage II patients or postmenopausal women are considered. Other factors such as progesterone receptors (McGuire *et al.*, 1986), thymidine labelling index (Silvestrini *et al.*, 1984) and S-phase fraction (McGuire & Dressler 1985), may predict disease-free survival.

Recently the epidermal growth factor receptor (EGF-R) was shown to be positively associated with high grade, ER −ve carcinomas and has been suggested as an additional cell marker to further segregate prognostically relevant breast cancer subsets (Sainsbury *et al.*, 1987).

We extended the search for biologically relevant markers in breast cancer and focused on two unrelated cell components of putative interest, the p53 protein and the cytoskeletal protein vimentin, after an extensive screening of several well characterized and new monoclonal antibodies.

p53 is a transformation-associated protein which has been included in the group of nuclear oncogenes because of its ability to immortalize normal cells and transform them in cooperation with a mutated oncogene (Eliyahu *et al.*, 1984). Vimentin, a class III intermediate filament (IF), is distributed on all mesenchymal cells and is coexpressed with other intermediate filaments on an increasing variety of epithelial tumours (Gould, 1985). Its presence on breast cancer cells was first described in pleural malignant effusions (Ramaekers *et al.*, 1983).

The function of vimentin is still unknown, but according to tentative explanation (Traub, 1985), vimentin is a nucleic acid-binding protein, regulated through a $Ca^{++}$ dependent proteinase, which is stored as polymerized filaments in the cytoplasm. The DNA-binding sequences of vimentin are analogous to the homologous region of the steroid hormone receptors (Traub, 1985).

In this study we show that p53, vimentin and EGF-R are expressed in Er −ve breast cancer cases and that they correlate positively with each other.

### Materials and methods

### *Antibodies*

PAb421 mouse anti-p53 monoclonal antibody (MAb) was kindly supplied as supernatant by Dr L. Crawford (Imperial Cancer Research Fund, London, UK). Anti-epidermal growth factor receptor R1 (M. Waterfield, ICRF, London, UK), anti-vimentin monoclonal (Amersham, Amersham, UK) and polyclonal antibodies (Euro BT, Roma, Italy) were also used. Both anti-vimentin antibodies gave identical reactivity on frozen sections of several types of normal and neoplastic tissues. They did not react with simple and stratified epithelia, skeletal muscle, most smooth muscle cells, parasympathetic ganglia and several tumour samples reported in the literature as negative for vimentin. A third anti-vimentin antibody (17βG3 from Ortho Diagnostic Systems, Raritan, NJ) crossreactive with GFAP, gave identical but weaker staining on the breast carcinomas tested, and was therefore excluded. The rabbit anti-vimentin antibody was used for the double labelling technique and for revealing the presence of vimentin on formalin-fixed, paraffin-embedded specimens stained for iconographic purposes. Breast cancer sections were stained for cytokeratins 8, 18 and 19 (MAb K4.62 from Bio-Yeda, Rehovot, Israel, and MAb UCD/PR 10.11 from Prof. R. Cardiff, UC Davis Medical Center, California, USA) with and without simultaneous vimentin staining. The rat anti-nuclear oestrogen receptor MAb (ER-ICA) was purchased from Abbott (Wiesbaden, FRG). Mouse MAbs directed against sheep red blood cells (Seralab, Crawley Down, UK) were used as negative controls.

All primary antibodies were used at saturating concentration after careful titration on appropriate targets and counterlabelled by indirect immunoalkaline phosphatase or immunoperoxidase methods as previously described (Cattoretti *et al.*, 1988). Double immunohistochemical staining was performed by first staining the nuclei for ER-ICA with the immunoperoxidase stain and then the cytoplasm for vimentin with Fast Blue BB immunoalkaline phosphatase stain. Double immunofluorescence was performed by incubating the slides with both the mouse anti-keratin and the rabbit anti-vimentin antibodies, and then by counterstaining with both the FITC anti-rabbit and the TRITC anti-mouse goat antibodies. The slides were then photographed with a Leitz epifluorescence microscope using Ilford HP4 B&W film.

### *Patients and tissues*

Fresh biopsy specimens from the operating theatre were snap-frozen in liquid nitrogen–cold isopentane (BDH, Poole, UK). A parallel sample was processed with routine techniques for morphological evaluation on paraffin sections.

Correspondence: G. Cattoretti.
Received 13 November 1987; and in revised form, 4 January 1988.

One hundred and niney-six primary breast carcinomas from 194 female and 2 male patients (aged 23–86 years; mean 53.04±0.797 s.e.) were collected: diameters ranged from 0.4 to 20 cm (median 1.9, mean 2.36±0.15 s.e.). One hundred and six were free of lymph-node metastasis, 33 had up to two positive nodes and 57 had three or more positive lymph nodes.

*Immunohistochemistry*

Immunohistochemical evaluation was performed on 4 $\mu$m thick frozen sections placed on clean glass slides and fixed in acetone or 10% buffered formaline (ER-ICA).

EGF-R and vimentin were considered positive when 10% or more of the tumour cells were stained. P53 and ER-ICA staining were considered positive when neoplastic cells exhibited unambiguous nuclear staining irrespective of the percentage of the positive cells. PAb421 antibody weakly cross-reacts with cytoplasmic cytokeratins and therefore nuclear staining only was evaluated (Cattoretti *et al.*, 1988).

Oestrogen (ER) and progesterone (PgR) receptors were measured simultaneously by a dual label dextran-coated charcoal (DCC) adsorption assay, previously described (DiFronzo *et al.*, 1986), by a single laboratory (Division of Experimental Oncology 'C', INT, Milano) on 132 unselected samples from 196 cases. Histologic typing was performed according to the guidelines recommended by the WHO and the histologic grading of the tumours was evaluated according to Bloom and Richardson (1957).

### Results

The analysis of 196 primary breast cancer specimens for p53, vimentin (VIM) and EGF-R showed that 15.8%, 25% and 34.2% respectively were positive with the appropriate antibody on tumour cells (Table I).

p53 (MAb PAb421) stained the nucleus of neoplastic (Figure 1a) but not normal epithelial cells (2 fibroadenomas, 2 lactating breast specimens, 6 mammary dysplasias and normal residual tissue surrounding breast carcinomas). Positive tumours were stained uniformly throughout the sections.

VIM +ve breast cancer cells had cytoplasmic fibrillary streaks of variable intensity (Figure 1b). The staining was irregulary distributed on each specimen. Double staining for VIM and keratin (Figure 2) and for VIM and ER (Figure 3) confirmed that the positive cells were in fact neoplastic epithelium. Connective tissue cells and lymphocytes were positive for vimentin in all specimens tested.

EGF-R was revealed by an exclusive membrane positivity (Figure 1c) and was homogeneously expressed on the totality of the tumour cells in most specimens.

The immunohistochemical ER staining (ER-ICA) was found on the nucleus of neoplastic cells and also in residual normal breast ductal cells; tumour heterogeneity was observed even though clearly negative and positive cases could be identified (see also DiFronzo *et al.*, 1986).

p53, VIM and EGF-R were distributed preferentially on ER-ICA −ve carcinomas (Table I).

As expected, p53, VIM and EGF-R reactivity largely (but not completely) overlapped on ER −ve specimens: 40/91 tumours found positive with one marker displayed an additional one or two antigens (Table II) and 36/45 (80%) ER-ICA −ve cases were positive for at least one marker *versus* 55/151 (36.4%) ER-ICA +ve ones ($\chi^2=28.92$, $P<0.001$).

Any combination of p53, VIM and EGF-R was significantly associated with ER-ICA −ve tumours (not shown), although the addition of p53 to VIM or EGF-R or the combination of both greatly reduced the absolute figures for ER-ICA coespression (Table II).

p53 alone or in combination seemed to be the major source of this effect, probably because it was found to be more restricted to the ER −ve tumours. Therefore, its contribution to the relationship of VIM and EGF-R with ER-ICA was assayed by evaluating the tumour panel without the p53 +ve cases; after removing the effect of p53, VIM and EGF-R still stained significantly more ER-ICA −ve carcinomas or tumours with less than 50% positive nuclei (Table III).

In 9 cases (4.59%) none of the determinants was expressed.

Oestrogen and/or progesterone receptors, determined biochemically, were significantly lower on p53 +ve (ER and PgR) and EGF-R +ve (PgR) carcinomas (Table IV).

p53, VIM and EGF-R +ve carcinomas were evaluated for the known parameters used to stage breast cancer; lymph node status was unrelated to the presence of the above cited markers, irrespective whether negative *vs* positive or 0–3 positive *vs.* >3 positive nodes was considered (not shown). The mean tumour diameter was greater in VIM +ve carcinomas (2.9±0.48 cm *vs.* 2.2±0.12 cm, $P<0.03$) and in EGF-R +ve ones (2.6±0.32 cm *vs.* 2.1±0.1 cm; $P=0.06$).

p53 and VIM +ve infiltrating duct carcinomas were more frequently scored as grade III (14/20 p53 +ve *vs.* 24/74 p53 −ve, $\chi^2=9.24$, df 2, $P=0.01$; 16/26 VIM +ve *vs.* 25/73 VIM −ve, $\chi^2=4.81$, df 1, $P<0.03$).

### Discussion

In this report we show that three unrelated cell proteins (nuclear p53, cytoplasmic vimentin and membrane EGF-R) cluster together and are expressed on hormonally defined subgroups of breast carcinomas.

Apart from the well-regulated distribution of vimentin in normal tissues and their malignant counterparts (reviewed by Gould, 1985), the *de novo* expression of this IF, together with other constitutive cytoskeletal proteins, has been the subject of extensive analysis. Two papers describe vimentin in breast carcinomas: one shows its presence in single cells in body fluids (Ramaekers *et al.*, 1983) and another, in a subset of breast and non-breast carcinomas (Azumi & Battifora, 1987). The coexpression of keratin and vimentin IF in epithelial malignancies has been associated with alterations is the shape and motility of the transformed cells (Henzen-Logmans *et al.*, 1987).

The *de novo* expression of vimentin during *in vitro* culture of epithelial cells has been shown to be a function of culture conditions (Ben-Ze'Ev, 1985; Rheinwald *et al.*, 1986) and the nutrients supplemented, with special reference to the hormones (hydrocortisone, insulin and prolactin) added to the media (Schmid *et al.*, 1983). Cells grown in hormone-depleted sera expressed vimentin IF whereas they did not when supplemented culture medium was added (Schmid *et al.*, 1983). It is not therefore unexpected to find the

**Table I** Expression of vimentin, p53 and EGF-R on ER-ICA positive and negative breast cancer

| | *Total cases* (%) | *ER-ICA −ve* (%) | *ER-ICA +ve* (%) | $\chi^2$ | *P* |
|---|---|---|---|---|---|
| Vimentin | 49/196 (25) | 26/45 (57.8) | 23/151 (15.2) | 31.23 | <0.001 |
| p53 | 31/196 (15.8) | 23/45 (51.1) | 8/151 (5.3) | 51.26 | <0.0001 |
| EGF-R | 67/196 (34.2) | 27/45 (60) | 40/151 (26.5) | 15.84 | <0.001 |

**Figure 1** (a) PAb421-positive breast cancer. Nuclear positivity is present on tumour cells but not on stromal cells (arrows). APAAP staining on frozen section (×600); (b) Vimentin-positive breast cancer. Tumour cells are labelled (arrows). ABC staining on formalin-fixed, paraffin-embedded specimen (×320); (c) EGF-R positive breast cancer. Membrane positivity is shown on tumour but not on stromal cells. APAAP staining on frozen section (×270). Scale bar = 5 μm.

**Figure 2** (a) Vimentin-positive breast cancer. Anti-cytokeratin antibody UCD/PR 10.11 selectively stains the epithelial cells, but not the stroma. TRITC immunofluorescence (×500); (b) Vimentin positive breast cancer. Polyclonal anti-vimentin antibody stains both the stroma and the tumour cells (asterisks). The staining pattern of vimentin on epithelial cells partially overlaps the cytokeratin staining but is more intense around the nucleus. FITC immunofluorescence. Same field as in (a) (×500). Scale bar = 5 μm.

**Figure 3** Double immunoenzymatic stain for ER-ICA (brown nuclei in the original colour slide) and for vimentin (blue cytoplasmic stain in the original colour slide). Double stained tumour cells are shown (arrows). Note the weak nuclear ER-ICA staining, typical of vimentin-positive breast carcinomas. Vimentin-positive stromal cells and lymphocytes (double arrows) do not show nuclear ER-ICA staining (×625). Scale bar = 5 μm.

phenotypic traits of an exogenously hormone-deprived cell in a tumour that lacks the appropriate hormone receptor. Experimental evidence that VIM is selectively expressed in hormone-independent, oncogene-transformed epithelial cell lines has been recently reported (Agnor *et al.*, 1987).

The vimentin gene was cloned from serum stimulated hamster fibroblasts (Rittling *et al.*, 1986) and expression was shown to be regulated at the transcriptional level be serum-derived growth factors such as PDGF, but not EGF, insulin or platelet-poor plasma (Ferrari *et al.*, 1986). Vimentin and the *myc* gene are induced with similar kinetics in early $G_1$ phase (Rittling *et al.*, 1986).

p53, which has been included on the nuclear oncogene family because of its biological transforming activity (Eliyahu *et al.*, 1984), bears no homology with known proto-oncogenes; however, it resembles another gene (*myc*) as far as its transforming strength, subcellular location and activation kinetics in *in vitro* models are concerned (Bienz *et al.*, 1984). By contrast with *myc* and vimentin, the p53 gene is maximally expressed in the mid $G_1$ cell cycle phase (Rittling *et al.*, 1986). p53 may be a regulatory element for the induction or maintenance of replicative DNA synthesis (Braithwaite *et al.*, 1987; Mercer *et al.*, 1982).

p53 is readily expressed after mitogen stimulation of normal cells *in vitro* (Mercer & Baserga, 1985), but not on resting cells, nor was it detectable on various normal tissues, including breast and mammary dysplasia (Cattoretti *et al.*, 1988). The presence of EGF-R has already been shown to be associated with large, high-grade, ER −ve breast carcinomas (Sainsbury *et al.*, 1987). EGF-R is at the same time the receptor for a natural mitogen on epithelial cells (Taketani & Oka, 1983) and the cellular homologue of the viral oncogene *erb*-B (Downward *et al.*, 1984).

**Table II** Subsets of breast carcinomas defined by the combination of vimentin, p53, EGF-R and ER-ICA reactivity

| *VIM* | *p53* | *EGF-R* | | *ER-ICA −ve* | *ER-ICA +ve* | *Total* |
|---|---|---|---|---|---|---|
| + | − | − | | 2 | 12 | 14 |
| − | + | − | | 4 | 3 | 7 |
| − | − | + | | 4 | 26 | 30 |
| + | + | − | | 3 | — | 3 |
| + | − | + | | 7 | 9 | 16 |
| − | + | + | | 2 | 3 | 5 |
| + | + | + | | 14 | 2 | 16 |
| | | | Subtotal | 36 | 55 | 91 |
| − | − | − | | 9 | 96 | 105 |
| | | | Total | 45 | 151 | 196 |

**Table III** Expression of vimentin and EGF-R on p53 negative breast cancer subsets

| | *Vimentin +ve* (%) | *EGF-R +ve* (%) |
|---|---|---|
| Total | 30/164 (18.3) | 46/164 (28) |
| ER-ICA −ve | 9/22 (40.9) | 11/22 (50) |
| ER-ICA +ve <50%[a] | 11/49 (22.4) | 18/49 (36.7) |
| ER-ICA +ve >50%[a] | 10/93 (10.8) | 17/93 (18.3) |
| $\chi^2$ | 11.63 | 11.48 |
| *P* value | <0.003 | <0.003 |

[a]Percentage of tumour cells stained.

**Table IV** ER and PgR values in vimentin, p53 and EGF-R defined breast cancer subsets

| | *ER values ± s.e. (fmol mg⁻¹ protein)* | *Significance* |
|---|---|---|
| Vimentin +ve | 81.56 ± 26.54 | $t=1.32$, df 130, $P=0.18$ |
| Vimentin −ve | 127.15 ± 18.51 | |
| p53 +ve | 27.8 ± 7.96 | $t=2.43$, df 130, $P<0.05$ |
| p53 −ve | 130.23 ± 17.63 | |
| EGF-R +ve | 74.72 ± 18.26 | $t=1.92$, df 130, $P=0.053$ |
| EGR-R −ve | 136.10 ± 21.13 | |
| | *PgR values ± s.e. (fmol mg$^{-1}$ protein)* | *Significance* |
| Vimentin +ve | 139.17 ± 45.04 | $t=1.41$, df 130, $P=0.15$ |
| Vimentin −ve | 225.34 ± 33.1 | |
| p53 +ve | 67.2 ± 25.18 | $t=2.12$, df 130, $P=0.03$ |
| p53 −ve | 225.88 ± 31.2 | |
| EGF-R +ve | 107.89 ± 24.9 | $t=2.58$, dr 130, $P=0.01$ |
| EGF-R −ve | 252.09 ± 38.55 | |

Several pieces of experimental evidence indicate that the inverse relationship found between ER on the one hand and p53, VIM and EGF-R on the other, has some basis in the proliferative status of the tumour cell; breast cancer progenitor cells in culture lack ER (Kodama *et al.*, 1985) and rapidly proliferating cells accumulate a low amount of the receptor (Jakesz *et al.*, 1984). p 53 itself has been found to parallel the RNA content of cycling cells and the p53 intracellular content rises when the cell proceeds to the $G_{1b}-G_2$ phase (Darzynkiewicz *et al.*, 1986). p53 +ve carcinomas display significantly higher reactivity with the proliferation associated Ki-67 MAb and express a higher amount of the transferrin receptor (Cattoretti *et al.*, 1988). Similarly, the median percentage of S-phase cells is higher in tumours lacking both ER and PgR (McGuire *et al.*, 1986; McGuire & Dressler, 1985).

In addition, the presence of EGF-R may indicate the ability of the cell to be stimulated by natural mitogens such as EGF or TGFα, the latter also being able to partially replace the tumour promoting effect of oestrogen (Dickson *et al.*, 1986). The presence of EGF-R immunoreactivity has also been positively related to a high S-phase content of breast cancer (Walker & Camplejohn, 1986). We can thus speculate that the presence of either one of p53, VIM or EGF-R is an indicator of an actively cycling tumour cell with a reduced amount of ER or a diminished requirement of ER to grow.

Approximately half of the breast cancer specimens found positive for one marker were positive for another one or two, but all the possible combinations were represented as outlined in Table II. The large spectrum of phenotypes observed can be explained in two ways: either minimal amounts of the apparently negative cell marker could not be detected in some cases because of the limited sensitivity of the technique employed; or because the genes involved depend on coincident, but not identical, stimuli to be expressed and therefore the membrane phenotype is indeed heterogeneous. Although we are aware of the limitations of the very sensitive techniques we used, we favour the latter explanation. There is experimental evidence that *myc* and p53 respond differently to EGF (Filmus *et al.*, 1987), phytohemagglutinin and interleukin 2 (IL2) stimulation (Reed *et al.*, 1986). However, both are expressed in cycling cells together with the IL2 receptor and both are necessary for IL2-induced growth (Reed *et al.*, 1986). Vimentin, in contrast to *myc*, responds to PDGF but not to EGF.

There is also evidence that a tumour cell with a given growth factor receptor repertoire may produce multiple growth factors, even those for which it has no membrane receptors (Betsholtz *et al.*, 1987); furthermore, not all tumour cell lines produce the same variety of growth factors (Peres *et al.*, 1987).

If we assume that p53 and vimentin depend on two separate combinations of growth factors and receptors to be induced, then it is conceivable that coexpression of both in tumour cells reflects the frequent but not obligate coexistence of several autocrine pathways.

Despite all the caveats outlined above, we identified two well characterized proteins which definitely indicate the cellular phenotype of an ER negative breast carcinoma and thus may facilitate the search for the appropriate growth factors and receptors.

Whether the combined expression of EGF-R, vimentin and p53 contributes a growth advantage to the tumour causing a more aggressive, hormone-independent proliferation of a given carcinoma must still be determined by *in vitro* and *ad hoc* clinical studies.

This work was partially supported by the Associazione Italiane Ricerca Cancro.

Drs L.V. Crawford and M. Waterfield (ICRF, London, UK) graciously supplied the anti-p53 and anti-EGF-R monoclonal antibodies.

We wish to thank Dr G. DiFronzo for having made available his data on ER and PgR biochemical measurement of breast carcinomas, Prof. M.I. Colnaghi for helpful criticism, Mr M. Azzini for photographic work and Mrs M. Hutton for reviewing the manuscript.

### References

AGNOR, C., WALKER-JONES, D., VALVERIUS, E. & 6 others (1987). Vimentin expression characterizes hormone-independent breast cancer cell lines and mammary epithelial cells transformed by two oncogenes. *Proc. Am. Assoc. Cancer Res.*, **28**, 11 (Abstract 41).

AZUMI, N. & BATTIFORA, H. (1987). The distribution of vimentin and keratin in epithelial and nonepithelial neoplasms. *Am. J. Clin. Pathol.*, **88**, 286.

BEN-ZE'EV, A. (1985). Cell–cell interaction and cell configuration related control of cytokeratins and vimentin expression in epithelial cells and in fibroblasts. In *Intermediate filaments*, Wang *et al.* (eds) *Ann. N.Y. Acad. Sci.*, **445**, 597.

BETSHOLTZ, C., BERGH, J., BYWATER, M. & 8 others (1987). Expression of multiple growth factors in a human lung cancer cell line. *Int. J. Cancer*, **39**, 502.

BIENZ, B., ZAKUT-HOURI, R., GIVOL, D. & OREN, M. (1984). Analysis of the gene coding for the murine cellular tumour antigen p53. *EMBO J.*, **3**, 2179.

BLOOM, H.J.G. & RICHARDSON, W.W. (1957). Histological grading and prognosis in breast cancer. *Br. J. Cancer*, **11**, 359.

BRAITHWAITE, A.W., STURZBECHER, H.W., ADDISON, C., PALMER, C., RUDGE, K. & JENKINS, J.R. (1987). Mouse p53 inhibits SV40 origin-dependent DNA replication. *Nature*, **329**, 458.

CATTORETTI, G., RILKE, F., ANDREOLA, S., D'AMATO, L. & DELIA, D. (1988). p53 expression in breast cancer. *Int. J. Cancer*, **41**, 178.

DARZYNKIEWICZ, Z., STAIANO-COICO, L., KUNICKA, J.E., DELEO, A.B. & OLD, L.J. (1986). p53 content in relation to cell growth and proliferation in murine L1210 leukemia and normal lymphocytes. *Leukemia Res.*, **10**, 1383.

DICKSON, R.B., MCMANAWAY, M.E. & LIPPMAN, M.E. (1986). Estrogen-induced factors of breast cancer cells partially replace estrogen to promote tumor growth. *Science*, **232**, 1540.

DIFRONZO, G., CLEMENTE, C., CAPPELLETTI, V. & 5 others (1986). Relationship between ER-ICA and conventional steroid receptor assays in breast Cancer. *Breast cancer Res. Treat.*, **8**, 35.

DOWNWARD, J., YARDEN, Y., MAYES, E. & 6 others (1984). Close similarity of epidermal growth factor receptor and v-erb-B oncogene protein sequences. *Nature*, **307**, 521.

ELIYAHU, D., RAZ, A., GRUSS, P., GIVOL, D. & OREN, M. (1984). Participation of p53 cellular tumour antigen in transformation of normal embryonic cells. *Nature*, **312**, 646.

FERRARI, S., BATTINI, R., KACZMAREK, L. & 6 others (1986). Coding sequence and growth regulation of the human vimentin gene. *Mol. Cell. Biol.*, **6**, 3614.

FILMUS, J., BENCHIMOL, S. & BUICK, R.N. (1987). Comparative analysis of the involvement of p53, *c-myc* and *c-fos* in epidermal growth factor-mediated signal transduction. *Exp. Cell. Res.*, **169**, 554.

GOULD, V.E. (1985). The coexpression of distinct classes of intermediate filaments in human neoplasms. *Arch. Pathol. Lab. Med.*, **109**, 984.

HENZEN-LOGMANS, S.C., MULLINK, H., RAEMEKERS, F.C.S., TADEMA, T. & MEIJER, C.J.L.M. (1987). Expression of cytokeratins and vimentin in epithelial cells of normal and pathologic thyroid tissue. *Virchows Arch.*, **410**, 347.

JAKESZ, R., SMITH, C.A., AITKEN, S. & 4 others (1984). Influence of cell proliferation and cell cycle phase on expression of estrogen receptor in MCF-7 breast cancer cells. *Cancer Res.*, **44**, 619.

KODAMA, F., GREENE, G.K. & SALMON, S.E. (1985). Relation of estrogen receptor expression to clonal growth and antiestrogen effects on human breast cancer cells. *Cancer Res.*, **45**, 2720.

MCGUIRE, W.L. & DRESSLER, L.G. (1985). Emerging impact of flow cytometry in predicting recurrence and survival in breast cancer patients. *J. Natl Cancer Inst.*, **75**, 405.

MCGUIRE, W.L., CLARKE, G.M., DRESSLER, L.G. & OWENS, M.A. (1986). Role of steroid hormone receptors as prognostic factors in primary breast cancer. *J. Natl Cancer Inst. Monogr.*, **1**, 19.

MERCER, W.E. & BASERGA, R. (1985). Expression of the p53 protein during the cell cycle of human peripheral blood lymphocytes. *Exp. Cell. Res.*, **160**, 31.

MERCER, W.E., NELSON, D., DELEO, A.B., OLD, L.J. & BASERGA, R. (1982). Microinjection of monoclonal antibody to protein p53 inhibits serum-induced DNA synthesis in 3T3 cells. *Proc. Natl Acad. Sci. USA*, **79**, 6309.

PERES, R., BETSHOLTZ, C., WESTERMARK, B. & HELDIN, C.H. (1987). Frequent expression of growth factors for mesenchymal cells in human mammary carcinoma cell lines. *Cancer Res.*, **47**, 3425.

RAMAEKERS, F.C.S., HAAG, D., KANT, A., MOESKER, O., JAP, P.H.K. & VOOIJS, G.P. (1983). Coexpression of keratin- and vimetin-type intermediate filaments in human metastatic carcinoma cells. *Proc. Natl Acad. Sci. USA*, **80**, 2618.

REED, J.C., ALPERS, J.D., NOWELL, P.C. & HOOVER, R.G. (1986). Sequential expression of protooncogenes during lectin-stimulated mitogenesis of normal human lymphocytes. *Proc. Natl Acad. Sci. USA* **83**, 3982.

RHEINWALD, J.G., O'CONNEL, T.M., CONNEL, N.D. & 5 others (1986). Expression of specific keratin subsets and vimentin in normal human epithelial cells: a function of cell type and conditions of a growth during serial culture. *Cancer cells*, **1**, 217.

RITTLING, S.R., BROOKS, K.M., CRISTOFALO, V.J. & BASERGA, R. (1986). Expression of cell cycle-dependent genes in young and senescent WI-38 fibroblasts. *Proc. Natl Acad. Sci. USA*, **83**, 3316.

SAINSBURY, J.C., FARNDON, J.R., NEEDHAM, G.K. MALCOLM, A.J. & HARRIS, A.L. (1987). Epidermal-growth-factor receptor status as a predictor of early recurrence of and death from breast cancer. *Lancet*, **i**, 1398.

SCHMID, E., SCHILLER, D.L., GRUND, C., STADLER, J. & FRANKE, W.W. (1983). Tissue type-specific expression of intermediate filament proteins in a cultured epithelial cell line from bovine mammary gland. *J. Cell. Biol.*, **96**, 37.

SILVESTRINI, R., DAIDONE, M.G., BERTUZZI, A. & DIFRONZO, G. (1984). Relationship between estrogen and cellular proliferation. In *Recent results in cancer research: Clinical interest of steroid hormone receptors in breast cancer*, Leclercq, G. *et al.* (eds) p. 163. Springer-Verlag: Berlin.

TAKETANI, Y. & OKA, T. (1983). Biological action of epidermal growth factor and its functional receptor in normal mammary cells. *Proc. Natl Acad. Sci. USA*, **80**, 2647.

TRAUB, P. (1985). Are intermediate filament proteins involved in gene expression? In *Intermediate Filaments*, Wang, E. *et al.* (eds) *Ann. N.Y. Acad. Sci.*, **455**, 68.

WALKER, R.A. & CAMPELJOHN, R.S. (1986). DNA flow cytometry of human breast carcinomas and its relationship to transferrin and epidermal growth factor receptors. *J. Pathol.*, **150**, 37.